# Effects of microbial communities during the cultivation of three salt-tolerant plants in saline-alkali land improvement

**DOI:** 10.3389/fmicb.2024.1470081

**Published:** 2024-10-31

**Authors:** Yijun Wang, Huarui Gong, Zongxiao Zhang, Zeqiang Sun, Shenglin Liu, Changjian Ma, Xuejun Wang, Zhaohui Liu

**Affiliations:** ^1^State Key Laboratory of Nutrient Use and Management, Shandong Academy of Agricultural Sciences, Jinan, China; ^2^Key Laboratory of Agro-Environment of Huang-Huai-Hai Plain, Ministry of Agriculture and Rural Affairs, Shandong Academy of Agricultural Sciences, Jinan, China; ^3^Institute of Agricultural Resources and Environment, Shandong Academy of Agricultural Sciences, Jinan, China; ^4^Institute of Geographic Sciences and Natural Resources Research, Chinese Academy of Sciences, Beijing, China; ^5^College of Geographic Science and Tourism, Xinjiang Normal University, Ürümqi, China; ^6^National Center of Technology Innovation for Comprehensive Utilization of Saline-Alkali Land, Institute of Modern Agriculture on Yellow River Delta of Shandong Academy of Agricultural Sciences, Dongying, China

**Keywords:** severely saline–alkaline soil, agriculture, tall wheatgrass, chicory, alfalfa, soil quality assessment

## Abstract

Planting vegetation on saline-alkaline land enhances soil fertility and sustainability by improving salt-alkali tolerance. Different salt-tolerant plant species interact with soil microorganisms, enriching bacterial communities and promoting nutrient availability. In this study, mechanisms affecting microbial communities in severely saline-alkaline soils planted with salt-tolerant plants are investigated. Over 4 years, the potential to cultivate three salt-tolerant plant species (tall wheatgrass *Agropyron elongatum*, chicory *Chicorium intybus*, and alfalfa *Medicago sativa*) in severely saline-alkaline soils is compared with a non-cultivated control. Bacterial and fungal communities were characterized through high-throughput sequencing of the 16S rRNA gene V3–V4 region and the V4 region, respectively. Cultivating these three plant species significantly reduces soil electrical conductivity values. Chicory cultivation notably increased soil nutrients, bacterial alpha richness, and fungal alpha diversity and richness. Microbial community structures vary considerably between the control and treatments, significantly correlating with the soil quality index. This index enables an assessment of soil health and fertility by integrating variables such as nutrient content, microbial diversity, and salinity levels. In each plant treatment, particularly alfalfa, the relative abundances of fungal pathogens like *Neocosmospora* and *Gibellulopsis* increase, which may pose risks to subsequent crops such as tomatoes, requiring careful consideration in future planting decisions. Conversely, in alfalfa and tall wheatgrass treatments, there was an increase in the relative abundances of fungal genera (e.g., *Alternaria* and *Podospora*) that antagonize fungal pathogens, while Paraphoma increased in the chicory treatment. The strong relationship between microorganisms and the rise in pathogen-resistant fungi across different plant treatments highlights robust and beneficial structural characteristics. According to soil quality index scores, each treatment, but especially that of chicory, improved the severely saline–alkaline soil environment.

## 1 Introduction

Global declines in agricultural productivity are associated with climate change, global temperature rises, and increased environmental stresses (Hassani et al., 2021). Saline soils pose a major problem for agricultural development worldwide (Etesami and Noori, 2019). Negative effects of salt exposure include changes to soil functions, which lead to, for example, decreases in agricultural productivity, water quality, soil biodiversity, and soil erosion, all of which limit land-resource use (Liu et al., 2014; Wu et al., 2015; Shi et al., 2019). Phytoremediation, as a cost-effective and environmentally friendly technology, is widely recommended for transforming saline–alkaline land into agricultural land by improving soil quality (Jesus et al., 2015; Bharti et al., 2017). Planting salt-tolerant species can improve soil physical and chemical properties by increasing soil permeability, and lowering the water table. Halophytes can also absorb and use soluble salt ions in the soil, reducing soil salinity while increasing the number and diversity of soil microorganisms.

Planting vegetation on saline–alkaline land promotes soil fertility, sustainability, and self-maintenance. However, relatively few plant species can tolerate saline conditions, and it can take a long time to achieve the desired beneficial effects (Cao et al., 2014; Rathore et al., 2017; Jing et al., 2019). Salt affects soil in more than 100 countries. Globally, salinized soil not only accounts for ~25% of all land, but this land area expands annually by 10,000–150,000 km^2^ (Ashraf, 2009; Spano et al., 2013; Xia et al., 2019). Therefore, the selection of an appropriate vegetation type is critical for the effective conversion of saline–alkaline land into a land that is more suitable for agriculture.

Chicory (*Cichorium intybus*), alfalfa (*Medicago sativa*) and tall wheatgrass (*Agropyron elongatum*) are all highly tolerant to saline–alkaline conditions (Deutch and Winicov, 1995; Zhao et al., 2001; Arshi et al., 2010; Li et al., 2022). Chicory is cultivated throughout Europe, India, South Africa, and Chile (Street et al., 2013). Alfalfa is widely grown as forage because of its high yield and adaptability, particularly in temperate regions worldwide (Annicchiarico et al., 2015). Tall wheatgrass—a good option for growing biomass in cold deserts and arid lands—is also cultivated throughout Europe (Porensky et al., 2014). Of these species, *C. intybus* and *M. sativa* significantly alter the physical and chemical properties of saline–alkaline soils, rendering them more suitable for agriculture (Liu et al., 2022). Tall wheatgrass, a high-yielding salt-tolerant grass, can produce fodder from its above-ground stems and leaves, and its root system can alter saline–alkaline soil (Dickeduisbeg et al., 2017; Zhu et al., 2020).

Numerous studies have shown that high salt levels in saline–alkaline soils can cause ion toxicity to plants and microorganisms, inhibiting organic matter accumulation and nutrient cycling (Lin et al., 2004; Tran et al., 2018; Yan et al., 2021). The interaction between microorganisms and plants significantly affects plant salt–alkali tolerance and the effectiveness of improving the quality of severely saline–alkaline soils. Different salt-tolerant plant species also have varying salt–alkali adaptation abilities, which can selectively recruit and enrich soil bacterial members, promote microbial activity, and enhance soil nutrient availability (Dumbrell et al., 2010; Ofek-Lalzar et al., 2014). Therefore, the interactions between soil microorganisms and plants may facilitate the improvement of soil quality to make it more suitable for agricultural use (Jing et al., 2019). However, there remains a knowledge gap in evaluating the remediation potential of different salt-tolerant plants on severely saline–alkaline soils, as well as the understanding of microbial community responses to plant remediation on marginal lands, particularly severely saline–alkaline soils. Thus, we explore the effects of planting three widely distributed and internationally cultivated plant species (chicory, alfalfa, and tall wheatgrass) on soil quality in saline–alkaline lands, and their interactions with soil microorganisms. While chicory and alfalfa are commonly referred to as plants, and tall wheatgrass as a grass, for convenience we refer to all three as plants hereinafter. Results and their interpretation provide a scientific basis and strategy for the global use of plants to improve soil quality in saline–alkaline lands for agricultural purposes.

## 2 Materials and methods

### 2.1 Field conditions, plant varieties, and experimental design

The study was performed at the Yellow River Delta Research Center, Chinese Academy of Sciences, Dongying City, Shandong Province (37°40′N, 118°55′E). A detailed site map for the experimental plot is provided in Supplementary Figure 1. The study area features a warm temperate monsoon climate with an annual mean temperature of 12.6°C. Precipitation varies throughout the year, with an average annual rainfall of ~556.1 mm, with 75% of this occurring between June and September. Soils are predominantly silty loams of pH 8.5, 1:2.5 w/v ratio of soil: water, organic matter 11.05 g kg^−1^, total nitrogen 0.60 g kg^−1^, Olsen-P(AP) 1.35 mg kg^−1^, available potassium 167.5 mg kg^−1^, and salinity 0.23%.

Three salt-tolerant plant species (tall wheatgrass, chicory, and alfalfa) were trialed. Plant rotation planting began in 2020. Trials were conducted in 6 × 9 m plots (54 m^−2^). Twelve plots (1 control, 3 treatment, × 3 replicate each) were spaced 60 cm apart. Each plant species was planted separately into three plots that were randomly distributed throughout the experimental area. In addition to a control (unplanted treatment) there were three treatments (plots containing tall wheatgrass, chicory, or alfalfa). Plants were cut twice a year; the cut plant material was harvested, and then returned to the soil at a later date. Nitrogen (urea) and phosphorus (calcium superphosphate) fertilizers were applied separately each year in late March, late June, and late October, at annual application rates of 320 kg N ha^−1^ and 160 kg P_2_O_5_ ha^−1^. Each year, the first fertilization was applied by strip application in mid-to-late March. The first cutting was performed in early June, followed by top-dressing fertilization. The second cutting was done in late October, followed by another round of top-dressing fertilization.

### 2.2 Soil sampling and analysis

#### 2.2.1 Soil sample collection

Soil samples were collected from 0 to 20 cm depth using a sterile blade in early March 2023 in a five-point sampling method. Each plot yielded 500 g of soil, with samples from each plot combined to create a composite sample. After visible plant root and organic residue material were removed, one soil subsample was placed into a plastic container and transported to the laboratory in an ice-box, then stored at −80°C for microbial community analysis. A second subsample was air-dried at room temperature for soil physicochemical analysis.

#### 2.2.2 Soil physicochemical properties

The pH, electrical conductivity (EC), AP, and available K (AK) were determined by air-drying and sieving soil samples through an 8-mesh sieve. Alkali hydrolyzed nitrogen (AN) and soil organic matter (SOM) concentrations were measured by grinding soil samples and passing them through a 100-mesh sieve. Soil pH and EC were measured using a pH meter (SevenExcellence, Mettler-Toledo, China) and an EC meter with a 1:2.5 soil-to-water ratio. Alkali hydrolyzable nitrogen (AN) was detected using an alkaline hydrolysis diffusion method (Mulvaney and Khan, 2001). SOM was determined by a potassium dichromate sulfuric acid oxidation method (Ciavatta et al., 1991). AP was determined following Murphy and Riley (1962), and AK following Walker and Barber (1962).

#### 2.2.3 DNA extraction and high-throughput sequencing

Soil DNA was extracted from 0.5 g of freeze-dried soil using the E.Z.N.A. Soil DNA Kit (Omega Bio-tek, Norcross, GA, USA) following manufacturer protocols. DNA concentration and purity were assessed using a 1% agarose gel and a NanoDrop 2000 UV-vis spectrophotometer (Thermo Scientific, Wilmington, USA). The V3–V4 region of the 16S rRNA gene was amplified with primer pairs 338F and 806R, and the V4 region of the 18S rRNA gene was amplified using the SSU0817F and 1196R primer pair. PCR was conducted in triplicate with an ABI GeneAmp 9700 PCR thermocycler (ABI, CA, USA) with an initial denaturation at 95°C for 3 min, followed by 27 cycles (95°C for 30 s, 55°C for 30 s, 72°C for 30 s), a single extension at 72°C for 10 min, and ending at 4°C. After amplification, 3 μl of PCR product underwent 2.0% agarose gel electrophoresis and subsequent purification using an AxyPrep DNA Gel Extraction Kit (Axygen Biosciences, Union City, CA, USA). PCR product quantity was determined using a Quantus Fluorometer (Promega, USA).

Purified amplicons were sequenced on an Illumina MiSeq platform (Illumina, San Diego, USA) at Majorbio Bio-Pharm Technology Co., Ltd, Shanghai, China. QIIME (v 1.9.1) was used for demultiplexing, quality filtering, and processing of raw FASTQ files. Sequences with ≥97% similarity were assigned to the same operational taxonomic units (OTUs) with UPARSE software (v 7.1; http://drive5.com/uparse/). The Ribosomal Database Project (RDP) classifier was used against the SILVA 16S rRNA database for bacteria (Quast et al., 2013), and the 18S rRNA database for fungi (Amato et al., 2013). On average, each sample yielded 46,026 quality 16S sequences and 68,464 quality 18S sequences, with an average 418 bp read length for bacteria and 248 bp for fungi.

#### 2.2.4 Data and statistical analyses

Membership values for each environmental variable were calculated using membership functions, and data were standardized through factor analysis. Principal component analysis was then performed on standardized values (Dou et al., 2022) to calculate the Soil Quality Index (*SQI*). A higher value indicates better overall soil quality.

The type of membership function (ascending or descending) was determined based on whether the correlation between soil quality variability and each environmental variable value was positive or negative; membership values of each environmental indicator were calculated separately (Tian et al., 2020).

The ascending distribution function was calculated by Equation 1:


(1) $$\begin{array}{l} F\left(x_{i}\right)=\frac{\left(x_{ij}-x_{i\min}\right)}{\left(x_{i\max}-x_{i\min}\right)} \end{array}$$

The descending distribution function was calculated by Equation 2:


(2) $$\begin{array}{l} F\left(x_{i}\right)=\frac{\left(x_{i\max}-x_{ij}\right)}{\left(x_{i\max}-x_{i\min}\right)} \end{array}$$

In the formula: *x*_*ij*_ represents the measured value of a certain soil indicator after planting a specific plant species, and *x*_*i*max_ represents the maximum value of the i-th indicator and *x*_*i*min_ the minimum value of that indicator.

The weights and quality indices of soil quality indicators in plant treatments were calculated following Hu et al. (2020), by Equations 3, 4:


(3) $$\begin{array}{l} W_{i}=C_{i}/\overset{n}{\underset{i=1}{\sum }}C_{i} \end{array}$$

(4) $$\begin{array}{l} SQI=\overset{m}{\underset{j=1}{\sum }}K_{j}\left\{\overset{n}{\underset{i=1}{\sum }}W_{i}\times f\left(x_{i}\right)\right\} \end{array}$$

where *W*_i_ represents the weight of the i-th soil quality evaluation indicator in a certain principal component; *C*_i_ represents the absolute value of the factor loading of the i-th soil quality evaluation indicator in a certain principal component; *n* represents the number of soil quality evaluation indicators; *m* represents the number of principal components (= 4); *K*_i_ represents the variance contribution rate of the i-th principal component; and f(x_i_) represents the membership value of the i-th soil quality evaluation indicator.

Microbial alpha diversity, including richness indices (Chao1 index), diversity indices (Shannon index), and Peilou's evenness indices, was computed using the “vegan” package in R (v 4.0.5), as was Principal Coordinates Analysis (PCoA) and Redundancy Analysis (RDA). The Circos graph was built using Circos software (Krzywinski et al., 2009). A one-way analysis of variance (ANOVA) for each trait was performed, fitting the linear mixed model with fixed-effects tested for treatment, and replication in JMP^®^Pro v 16 (SAS, USA). The Tukey HSD test was applied in comparison with each other treatment following ANOVA to determine if significant differences existed between their means. Spearman's two-tailed correlation tests were performed using SPSS software v 25.0. The top 100 bacterial and fungal OTUs were filtered based on abundance; WGCNA (v1.72-5) was used to calculate Pearson correlation coefficients between OTUs. A correlation with |*r*| > 0.9 and *p* < 0.05 was considered significant. The network was visualized using Gephi (v 0.9).

## 3 Results

### 3.1 Soil chemical properties

Compared with bare saline–alkaline bare land, SOM contents in plant treatments did not significantly differ (Table 1). However, the SOM content in the chicory treatment increased significantly by 42.32% compared with the tall wheatgrass treatment (*P* < 0.05). Compared with the control, the AN content in chicory and alfalfa treatments significantly increased by 40.12 and 18.30%, respectively, while the AK content in the chicory and tall wheatgrass treatments significantly increased by 53.47 and 14.28%, respectively (*P* < 0.05). Of all plant treatments, chicory had the highest AN and AK contents. The AP content in soils in tall wheatgrass, chicory, and alfalfa treatments was 10.49×, 10.30×, and 7.81× that of the control, respectively, with the AP content in the alfalfa treatment significantly lower than that in tall wheatgrass and chicory treatments. Compared with the control, the changes in soil pH in different plant treatments were not statistically significant, but EC was significantly reduced. The pH in the chicory treatment was significantly lower than that of the alfalfa treatment.

**Table 1:** Effect of different plant treatments on selected soil parameters.

**Treatment**	**SOM**	**AN**	**AP**	**AK**	**pH**	**EC**
	**(g**·**kg**^−1^**)**	**(mg**·**kg**^−1^**)**	**(mg**·**kg**^−1^**)**	**(mg**·**kg**^−1^**)**		**(mS m** ^−1^ **)**
CK	25.76 ± 2.27ab	50.09 ± 5.32c	1.52 ± 0.56c	172.67 ± 25.03b	8.37 ± 0.38ab	6.70 ± 1.94a
GF	24.69 ± 1.80b	44.04 ± 0.75c	15.95 ± 0.03a	197.33 ± 6.66b	8.41 ± 0.04ab	1.92 ± 0.05b
JA	35.14 ± 3.63a	77.19 ± 4.53a	15.64 ± 0.97a	265.00 ± 6.35a	8.27 ± 0.15b	1.95 ± 0.18b
CP	29.60 ± 3.56ab	65.71 ± 2.20b	11.87 ± 0.25b	117.33 ± 3.79c	8.51 ± 0.02a	1.81 ± 0.018b

Soil parameters: SOM, soil organic matter; AN, available nitrogen; AK, available potassium; AP, available phosphorus; EC, electrical conductivity.

Treatments CK, control (no plants); CP, alfalfa; GF, tall wheatgrass; and JA, chicory.

Different letters indicate significant differences at *p* < 0.05.

### 3.2 Soil bacteria and fungi community structure and diversity

**Figure 1:**
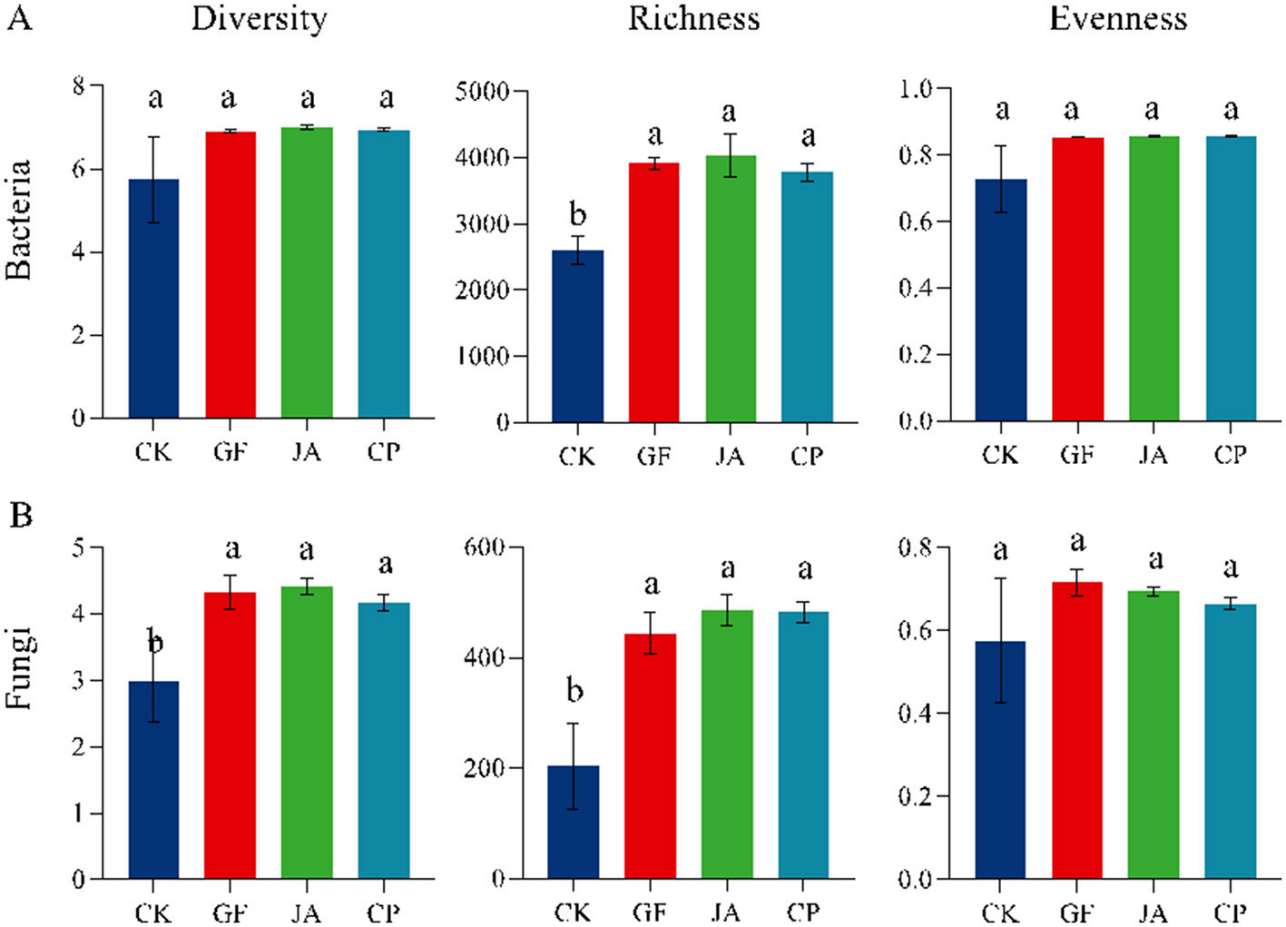
Alpha-diversities of soil bacteria **(A)** and fungi **(B)** in different plant treatments. The same lowercase letters are not significantly different at *p* < 0.05. Error bars represent standard deviations. Treatments CK, control (no plants); CP, alfalfa; GF, tall wheatgrass; and JA, chicory.

Bacterial and fungal richness index in treatments were significantly higher than those in the control (Figure 1; *P* < 0.05). Compared with the control, bacterial richness increased by 50.43, 55.09, and 45.24% in the tall wheatgrass, chicory, and alfalfa treatments, respectively, while fungal richness increased by 118.07, 138.91, and 136.95%, respectively (*P* < 0.05). Bacterial and fungal evenness did not change significantly between treatments. There were no significant differences in bacterial diversity among treatments, but fungal diversity in the tall wheatgrass, chicory, and alfalfa treatments increased significantly by 44.95, 47.82, and 39.57%, respectively, compared with the control (*P* < 0.05).

**Figure 2:**
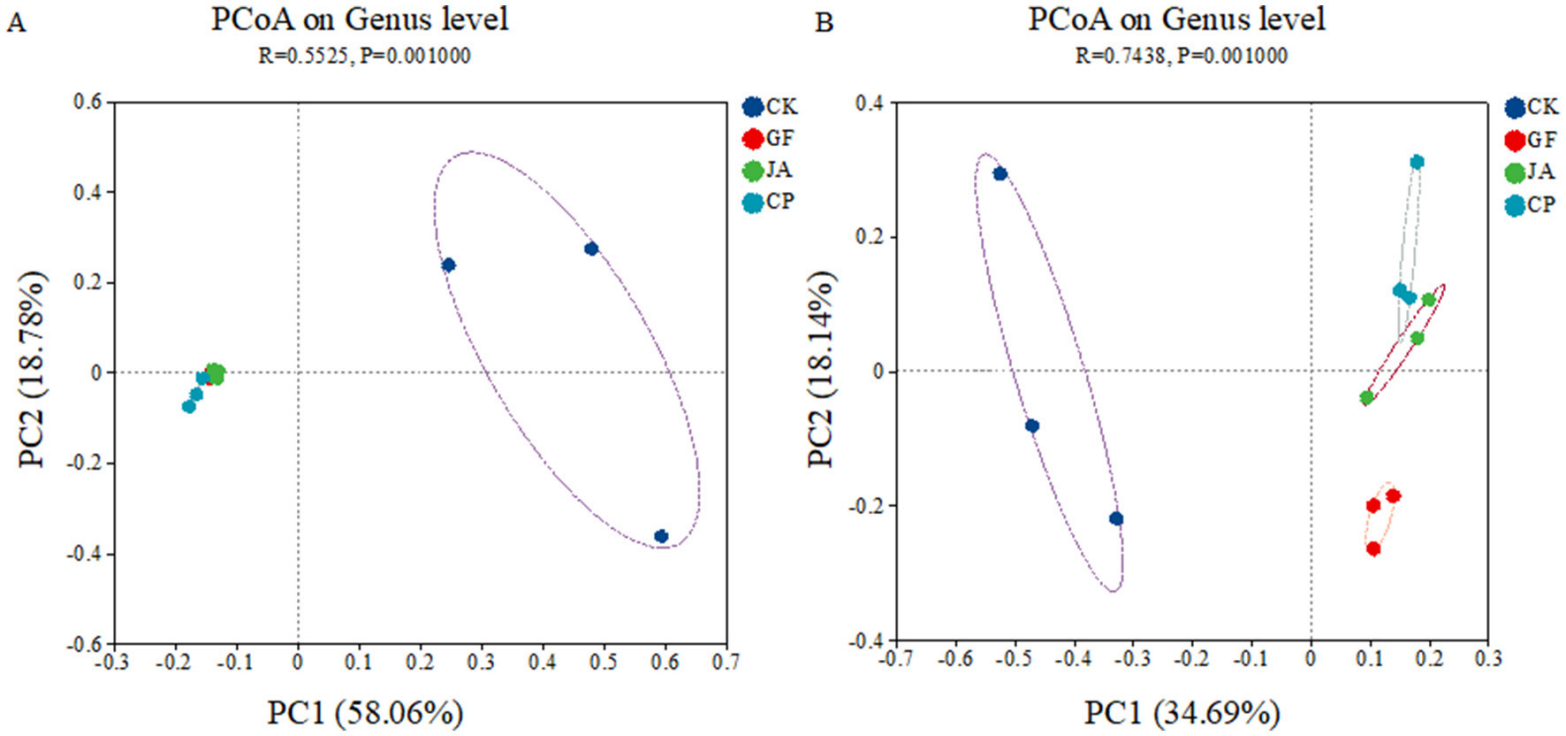
Principle coordinate analysis of Bray–Curtis distance for soil bacteria **(A)** and fungi **(B)**. Treatments CK, control (no plants); CP, alfalfa; GF, tall wheatgrass; and JA, chicory.

**Figure 3:**
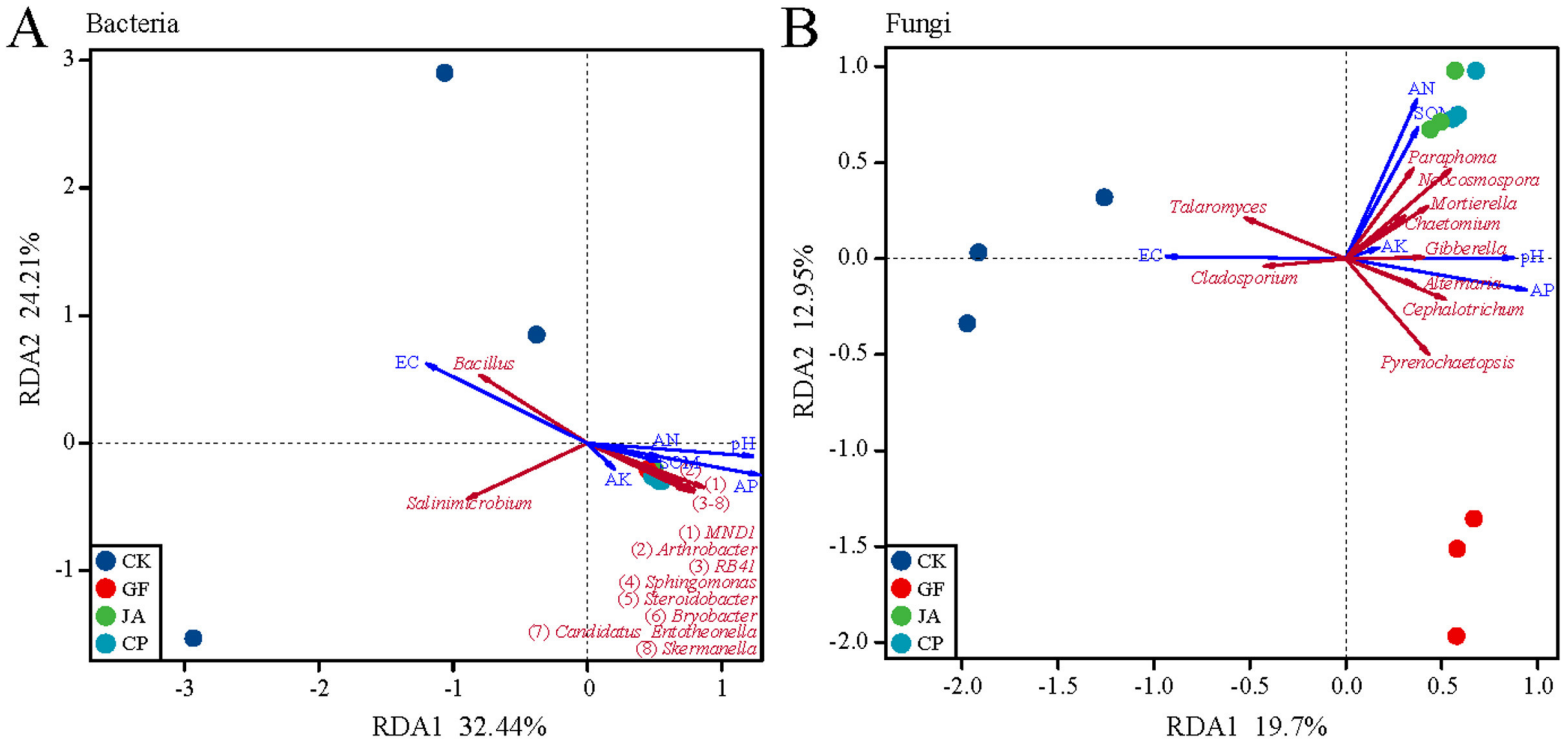
Redundancy analysis (RDA) ordination plots showing the relationship between soil environmental variables and microorganisims at bacteria genus **(A)** and fungal genus **(B)**, based on Bray–Curtis. Soil environmental variables: SOM, soil organic matter; AN, available nitrogen; AK, available potassium; AP, available phosphorus; EC, electrical conductivity. Treatments CK, control (no plants); CP, alfalfa; GF, tall wheatgrass; and JA, chicory.

The first two axes of the PCoA explained 58.06 and 18.78% of the variance in bacterial communities, and 34.69 and 18.14% of the variance in fungal communities, respectively. Bacterial communities in the control clearly separated from those in treatments, and fungal communities in treatments were well-separated on the second axis (Figure 2). RDA results show that the soil characteristics in treatments strongly influenced bacterial and fungal communities, with environmental indices explaining 74.46% of variation in the bacterial community and 50.66% in the fungal community (Figure 3). The negative correlation between EC and AP mainly affected variation in bacterial and fungal communities between the control and treatments. SOM and AN had a greater impact on fungal than bacterial communities. The correlation analysis of the top 10 bacterial/fungal genera with soil environmental factors revealed the presence of strong microbial species–environment relationships.

**Figure 4:**
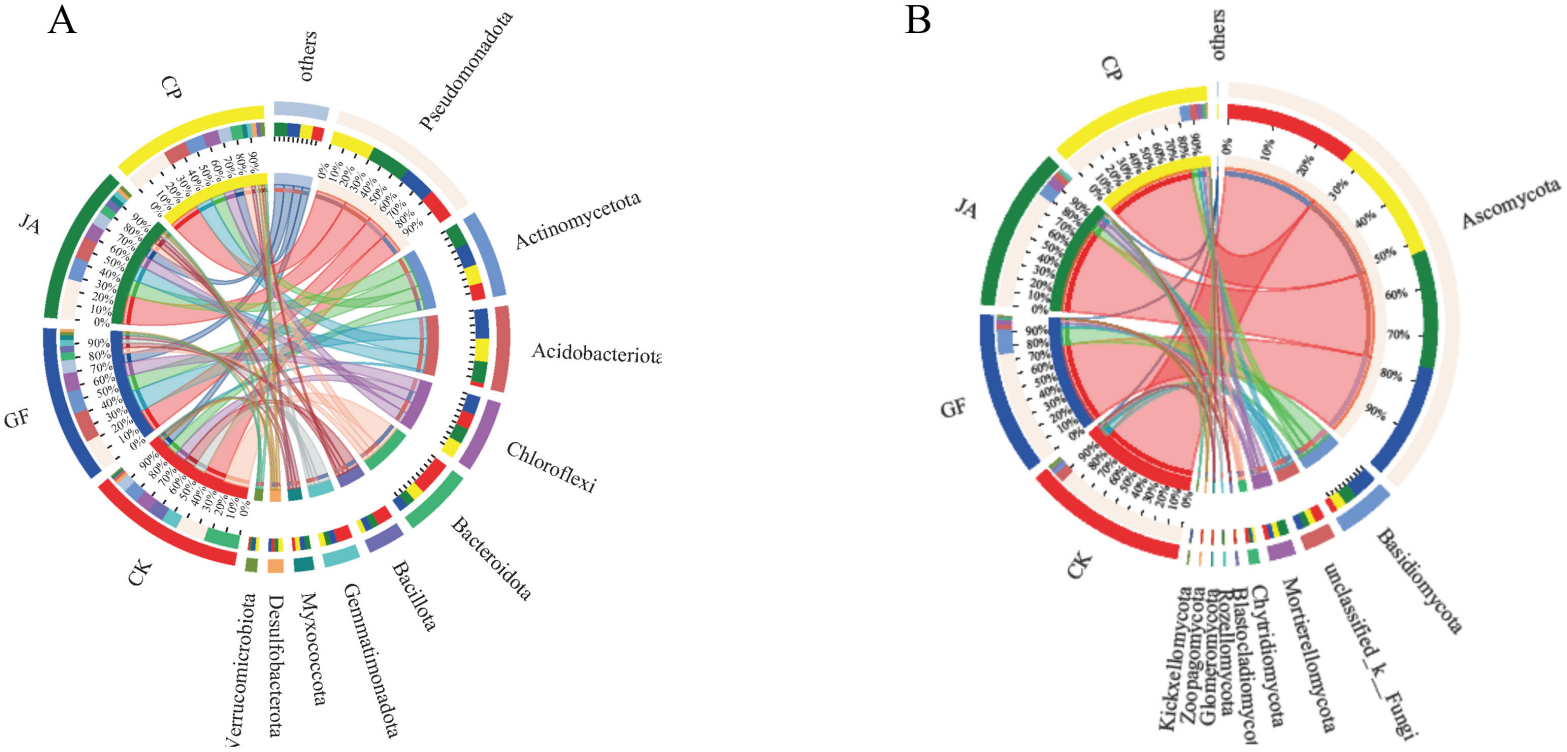
Circos plot of relationships between soil samples and bacterial **(A)** and fungal **(B)** species at the level of phylum. Treatments CK, control (no plants); CP, alfalfa; GF, tall wheatgrass; and JA, chicory.

Bacterial and fungal composition differed significantly among treatments (Figure 4A). The four dominant bacterial phyla were Pseudomonadota, Actinomycetota, Acidobacteriota, and Chloroflexi, with relative abundances in samples ranging 20–29%, 10–15%, 2–20%, and 11–13%, respectively; the relative abundances of other phyla were all <10%. Proportional abundances of phyla in CK, CP, JA, and GF treatments were Pseudomonadota (20, 30, 28, and 22%), Actinomycetota (19, 24, 29, and 28%), Acidobacteriota (4, 30, 28, and 39%), and Chloroflexi (24, 24, 24, and 29%), respectively. Compared with the control, the proportional abundances of Pseudomonadota, Actinomycetota, Acidobacteriota, Myxococcota, and Verrucomicrobiota increased significantly in treatments, while abundances of Bacteroidota, Bacillota, and Gemmatimonadota all decreased significantly. Verrucomicrobiota and Desulfobacterota were the dominant phyla in the alfalfa treatment, Myxococcota was dominant in the chicory treatment, and Acidobacteriota and Chloroflexi were dominant in the tall wheatgrass treatment.

The main fungal phyla in all samples were Ascomycota, Mortierellomycota, and Basidiomycota, with relative abundances ranging 75–88%, 11–42%, and 3–16%, respectively (Figure 4B). Compared with the control, the relative abundance of Ascomycota was significantly reduced, and those of Basidiomycota, Mortierellomycota, Chytridiomycota, and Zoopagomycota all significantly increased in treatments. The relative abundances of Chytridiomycota and Rozellomycota were highest in the alfalfa treatment; those of Mortierellomycota, Zoopagomycota, and Kickxellomycota were highest in the chicory treatment; and that of Basidiomycota was highest in the tall wheatgrass treatment.

### 3.3 Comprehensive evaluation of soil quality in plant treatments and effect of microbial community structure on soil quality

The contribution rates of the first four principal components were 57.37, 20.30, 18.77, and 4.28%, respectively, with a cumulative contribution rate of 95.73% (Supplementary Table 1). This indicates that the first four principal components explain most of the variation in soil quality. Based on positive and negative correlations of each environmental variable with soil quality, we selected the type of membership functions accordingly: soil SOM, AN, AP, AK, with the Chao1, and Shannon indices using ascending membership functions, and pH and EC using descending membership functions. The *SQI*, calculated from the weights of each soil indicator (Supplementary Table 1) and their membership degrees (Supplementary Table 2), revealed soil quality in each treatment exceeded that of the control (ranked chicory > alfalfa > tall wheatgrass > control; Table 2).

**Table 2:** Soil quality indices (*SQI*) and their order in treatments [CK, control (no plants); CP, alfalfa; GF, tall wheatgrass; and JA, chicory].

**Treatments**	***SQI***	**Order**
CK	22.49	4
GF	50.40	3
JA	84.13	1
CP	59.70	2

**Figure 5:**
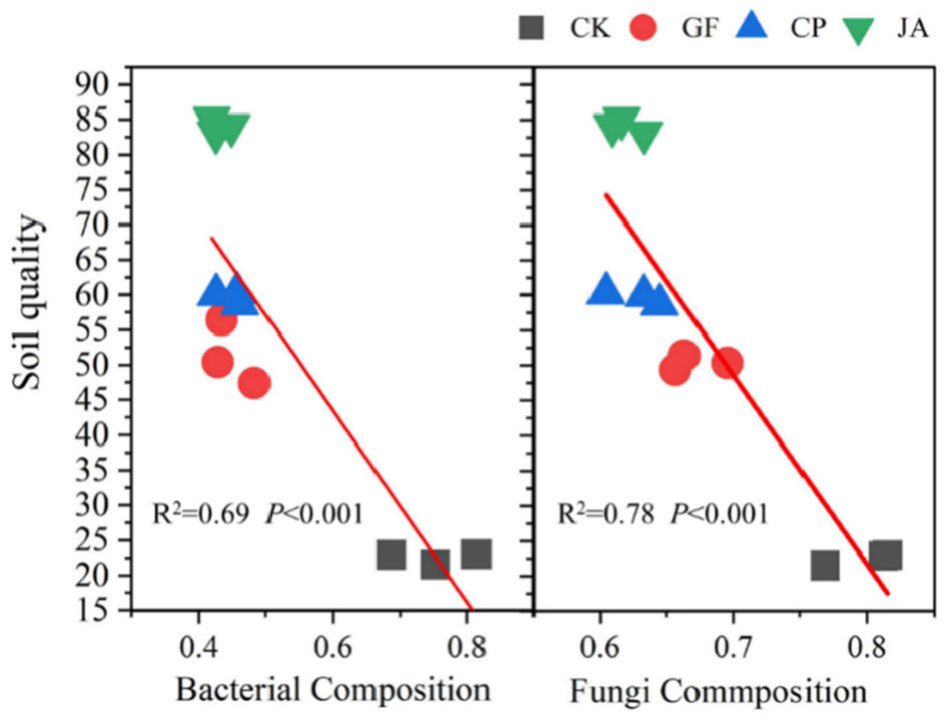
Regression of relationships between soil quality index and the Bray–Curtis distance of bacterial and fungal composition. Treatments CK, control (no plants); CP, alfalfa; GF, tall wheatgrass; and JA, chicory.

**Figure 6:**
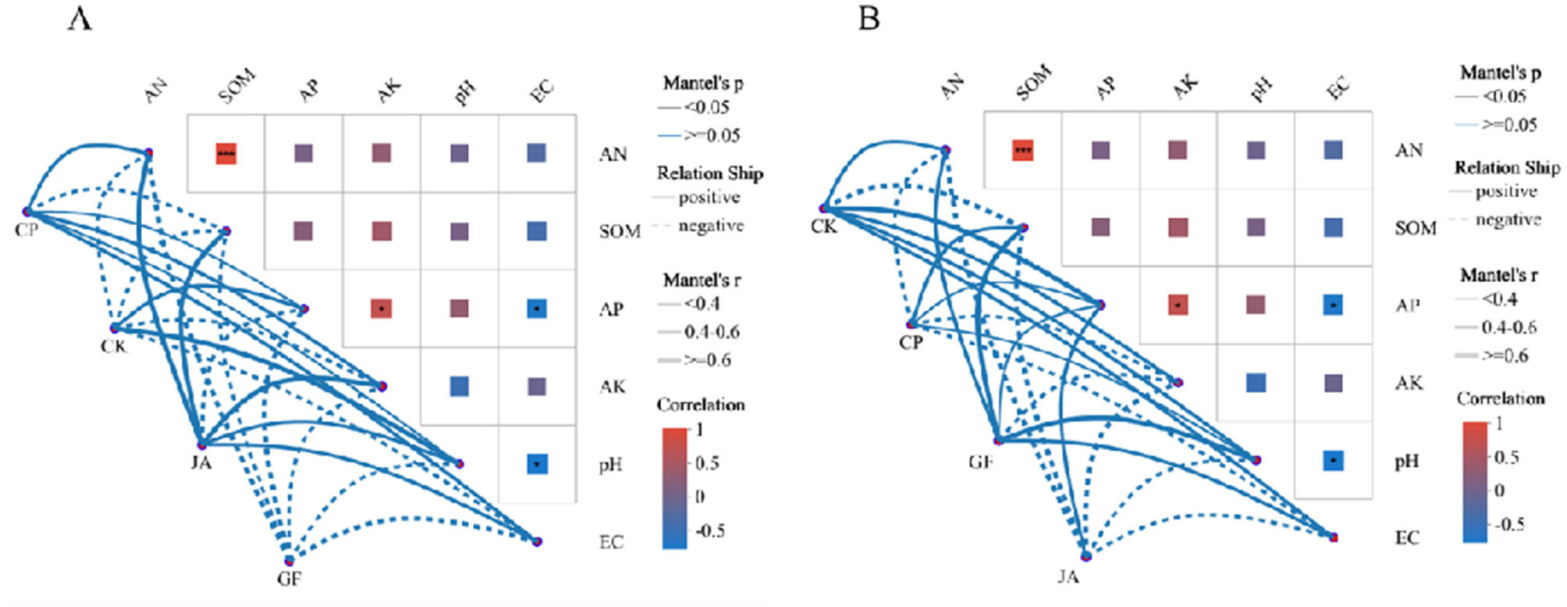
Mantel test showing soil factor correlations with bacterial **(A)** and fungal **(B)** communities. Edge width corresponds to absolute value of the correlation coefficient determined by the Mantel test. Colors indicate the magnitude of significant correlations. Treatments CK, control (no plants); CP, alfalfa; GF, tall wheatgrass; and JA, chicory.

Linear regression results (Figure 5) indicate that changes in soil bacterial and fungal community structure in forage cultivations are highly significantly correlated with *SQI*. Soil chemical factors driving bacterial and fungal community structures differ markedly in the control and CP treatments. In the control, bacterial community structure is only positively correlated with AP and pH, while the fungal community correlates positively with AN, AP, AK, pH, and EC. In the CP treatment, bacterial community structure correlates positively with AN, AP, AK, pH, and EC, while fungal community structure correlates positively with SOM, AP, and pH. In the JA treatment, the soil bacterial community structure is significantly, positively correlated with AN, SOM, AK, pH, and EC. In the GF treatment, soil fungal community structure is significantly, positively correlated with AN, SOM, AP, pH, and EC (Figure 6).

### 3.4 Significant differences in relative abundances of genera among soils cultivated with chicory, alfalfa, and tall wheatgrass, and correlation analysis of these genera with soil properties

**Figure 7:**
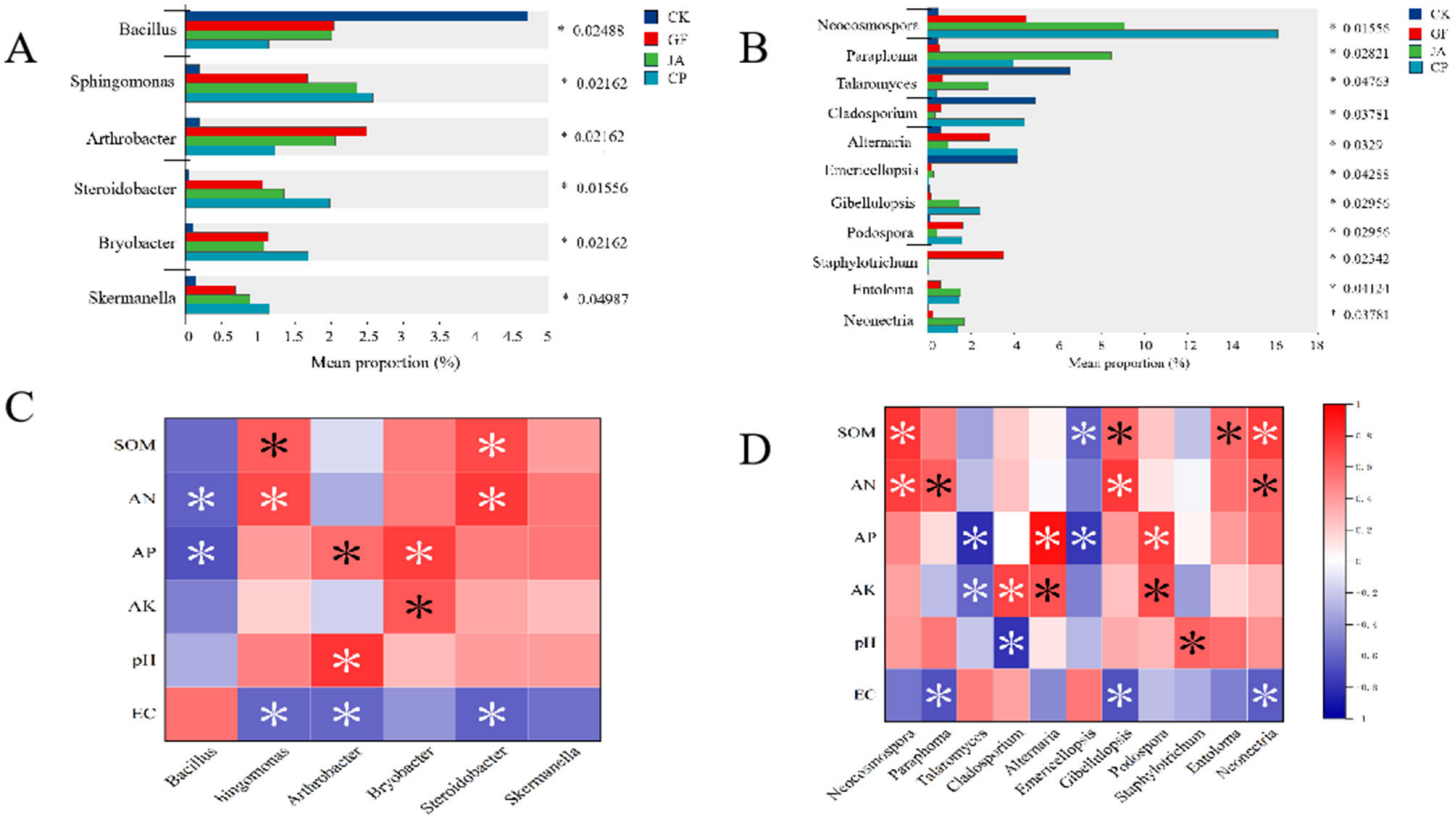
Relative abundances of bacteria genera **(A)** and fungi genera **(B)** that differed significantly among soil samples cultivated with chicory, alfalfa, and tall wheatgrass. A one-way ANOVA was used to evaluate the significance of differences between indicated groups. *P < 0.05. Correlation analysis of the relative abundance of bacteria **(C)** and fungi **(D)** genera with soil properties. Soil parameters: AN, available nitrogen; AK, available potassium; AP, available phosphorus; EC, electrical conductivity, SOM, soil organic matter. Treatments CK, control (no plants); CP, alfalfa; GF, tall wheatgrass; JA, chicory.

We compared the relative abundances of six bacterial and 11 fungal genera that differed significantly across treatments (Figure 7A). For bacteria, the relative abundance of *Bacillus* was significantly higher in the control than in treatments, whereas those of *Sphingomonas, Arthrobacter, Steroidobacter, Bryobacter*, and *Skermanella* were significantly lower in treatments. Among all treatments, *Sphingomonas, Steroidobacter, Bryobacter*, and *Skermanella* were relatively most abundant in the alfalfa treatment. Relative abundances of *Sphingomonas* and *Steroidobacter* correlated significantly and positively with SOM and AN, and significantly and negatively with EC; that of *Bryobacter* correlated significantly and positively with AP and AK. *Arthrobacter* was relatively most abundant in the tall wheatgrass treatment and correlated significantly and positively with AP and pH, and negatively with EC (Figure 7C).

For fungi, the relative abundances of *Talaromyces, Cladosporium*, and *Emericellopsis* were significantly higher in the control than in the treatments (Figure 7B). The relative abundance of *Emericellopsis* was significantly negatively correlated with SOM and AP; that of *Talaromyces* was significantly negatively correlated with AP and AK; and that of *Cladosporium* was significantly positively correlated with AP. In the alfalfa treatment, the relative abundances of *Neocosmospora, Alternaria*, and *Gibellulopsis* were the highest. Relative abundances of *Neocosmospora* and *Gibellulopsis* were significantly positively correlated with SOM and AN, while that of *Alternaria* was significantly positively correlated with AP and AK. In the tall wheatgrass treatment, the relative abundances of *Podospora* and *Staphylotrichum* were greatest, with that of *Podospora* being significantly positively correlated with AP and AK. In the chicory treatment, the relative abundances of *Paraphoma, Entoloma*, and *Neonectria* were greatest; that of *Entoloma* was significantly positively correlated with SOM; that of *Paraphoma* was significantly positively correlated with AN; and that of *Neonectria* was significantly positively correlated with SOM and AN. Relative abundances of both *Paraphoma* and *Neonectria* correlated negatively with EC (Figure 7D).

### 3.5 Co-expression network analysis

**Figure 8:**
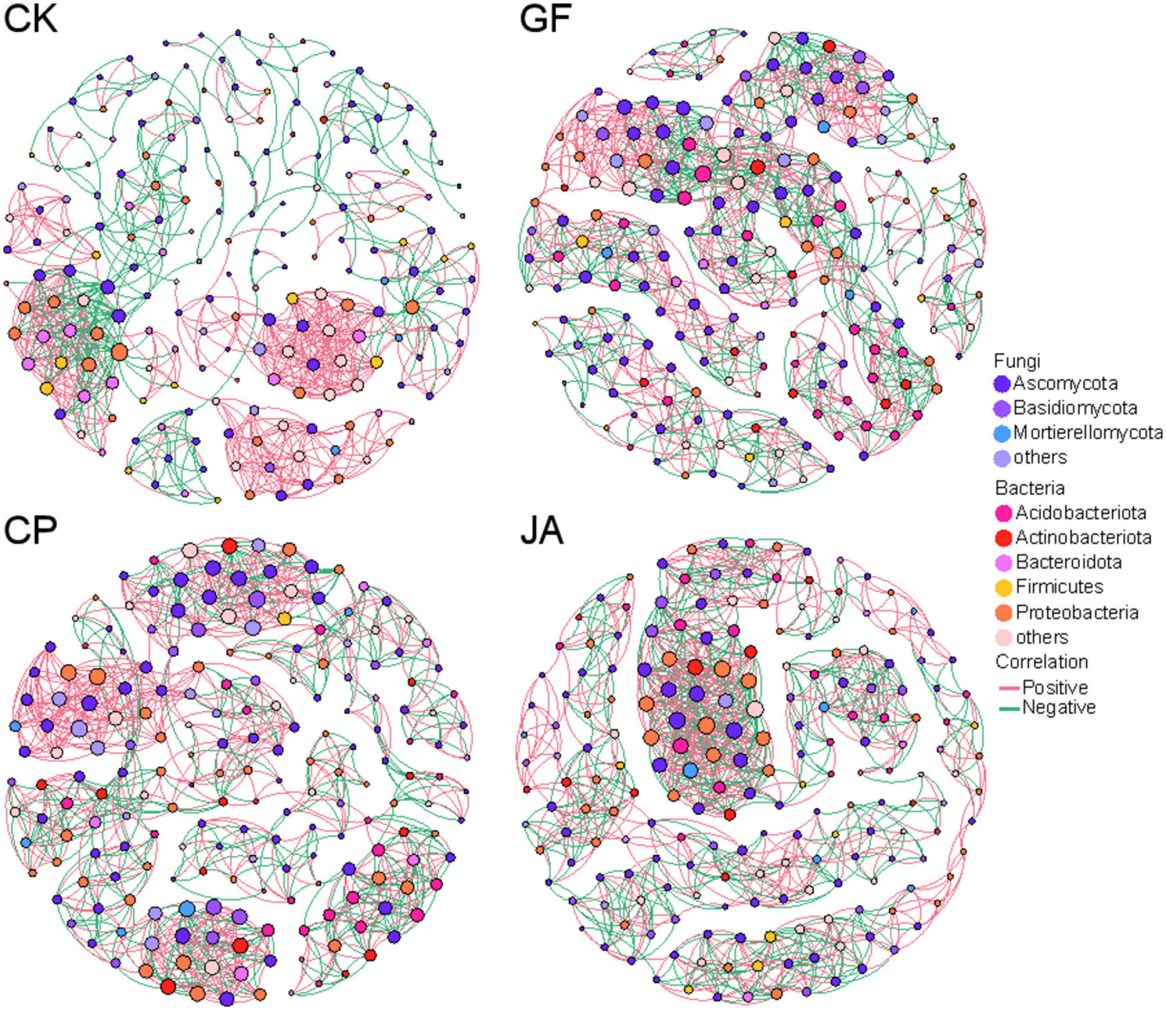
Network interactions between bacteria and fungi among treatments. CK, control (no plants); CP, alfalfa; GF, tall wheatgrass; and JA, chicory. The size of each node is proportional to the number of connections (i.e., degree).

To investigate co-expression patterns of bacteria and fungi in treatments, a correlation analysis was performed using Weighted Gene Co-Expression Network Analysis (WGCNA; Figure 8). Compared with the control, associations among microorganisms increased in each treatment, with numbers of positive correlations exceeding numbers of negative correlations, an increase of average degree, density, and average clustering coefficient (Table 3). Average path length in the JA treatment was highest among treatments. This indicates increased closeness, complexity, and relative stability in soil microbial associations. Compared with the alfalfa and tall wheatgrass treatments, the chicory treatment showed significantly more negative correlations with *Neocosmospora* and *Gibellulopsis*, for which both exceeded numbers of positive correlations. For each plant treatment, numbers of positive correlations for *Alternaria, Podospora*, and *Talaromyces* exceeded numbers of negative correlations (Supplementary Table 3).

**Table 3:** Number of linear links in networks for treatments [CK, control (no plants); CP, alfalfa; GF, tall wheatgrass; and JA, chicory].

	**CK**	**GF**	**JA**	**CP**
Total linear links	746	1,059	1,124	1,062
Positive links	478	529	607	647
Negative links	268	467	517	415
Average degree	7.54	10.58	11.23	10.61
Density	0.04	0.05	0.06	0.05
Modularity	0.82	0.81	0.79	0.84
Average clustering coefficient	0.65	0.76	0.74	0.77
Average path length	5.33	5.48	15.30	6.15

## 4 Discussion

### 4.1 Effect of cultivating three salt-tolerant plant species on soil improvement potential for saline–alkaline soils

Planting salt-tolerant species is a common approach to ameliorate saline–alkaline soils. However, most studies have focussed on the effects of a single halophyte species. In this study, the potential of three commonly used salt-tolerant plants for improving saline–alkaline soils was evaluated. Results demonstrate that these species can enhance soil nutrient content to varying degrees. Plants cultivation significantly reduced soil EC. Each plant can absorb and remove salt ions (Na^+^, Mg^2+^, and Ca^2+^) from saline–alkaline soils by increasing aboveground biomass (Shabala, 2013). Although the plants that absorbed soil salts were returned to the soil, the electrical conductivity (EC) did not increase; instead, it decreased significantly. Plant roots can enhance the leaching of soluble salts, thereby affecting the chemical environment of the soil, particularly by facilitating Na^+^ to Ca^2+^ exchange, which removes Na ions from the cation exchange sites in the soil (Robbins, 1986; Qadir et al., 1996). During the process of salt removal, leaching plays a more prominent role in removing Na^+^ from the soil compared to the contribution of removing Na^+^ through harvesting the shoot parts of the plant (Qadir et al., 2003). After cutting, cut vegetation was returned to the plot; being rich in organic matter, N was released into the soil over time. Additionally, cultivating salt-tolerant plants selectively attracts beneficial microorganisms through their root systems, which promotes the activation of soil nutrients and consequently increases the nutrient content of the soil (Egamberdieva et al., 2019). Of the three plants, the planting of chicory produced the highest levels of SOM, AN, AP, and AK, indicating that it was the most suitable of the three species to cultivate to improve soil conditions for agriculture in saline–alkaline land (Table 1).

### 4.2 Cultivation of three salt-tolerant plants affects microbial communities and relationships between these communities and soil physicochemical traits

The treatment of non-forage cultivation and the cultivation of different forage species caused significant differences in soil microbial community structures, indicating the strong selective pressure was exerted by forage cultivation on these microbial communities (Figure 2A). The bacterial phyla Pseudomonadota, Actinomycetota, and Acidobacteriota were dominant in each treatment. Actinomycetota (mostly saprophytic bacteria) have a strong ability to decompose complex macromolecular organic matter in soils (Jacquiod et al., 2013). Pseudomonadota play an active role in inducing plant disease resistance (Lee et al., 2021). Acidobacteria (mostly acidophilic bacteria) are involved in the soil N cycle and are closely related to soil organic matter content (Liu et al., 2016). This indicates that cultivation of certain plants can enhance carbon and N metabolism levels of soil microorganisms. The decreased relative abundance of bacterial phyla Bacillota and Gemmatimonadota benefits retention of organic matter (Zhang et al., 2014; Zhao et al., 2019; Liu et al., 2020). Different plants increased the relative abundances of saprophytic fungal phyla such as Basidiomycota, Mortierellomycota, and Chytridiomycota (but not Ascomycota), which assists with the decomposition of complex organic matter, accelerates net N mineralization rates, and promotes soil N cycling (James et al., 2006; Floudas et al., 2012; Tedersoo et al., 2018). The positive roles of beneficial bacterial and fungal communities indicate their contribution to changing saline–alkaline soil quality to render it more suitable for agricultural purposes. In the chicory treatment, Mortierellomycota was relatively most abundant. Mortierellomycota can solubilize phosphorus and form symbiotic relationships with plants. The abundance of Ascomycota does not correlate positively with soil health because it is also relatively abundant in diseased soils. The increased relative abundance of Mortierellomycota in soils may (partly) indicate greater soil health (Yuan et al., 2020).

Variation in soil microbial community structure is significantly positively correlated with soil quality (Figure 5). We report bacterial communities driven by the combined effects of AN, SOM, and AK under chicory cultivation to potentially play a significant role in improving soil quality, and for fungal communities driven by the combined effects of AN, SOM, and pH under tall wheatgrass cultivation to possibly significantly enhance soil quality. Biological N fixation is an important source of soil N in forage cultivation, and AN and SOM are key environmental factors that drive microbial community structure (Shao et al., 2021). The quantity and community structure changes of N-fixing microorganisms are important for soil N supply and fertility maintenance, thereby increasing microbial activity and promoting the turnover of organic matter and other soil nutrients (Blagodatskaya et al., 2009). Even though the cultivation of different forage species tends to increase the abundance of beneficial soil microorganisms, different plant genotypes and soil types play important roles in determining the types and quantities of microbial species (Marschner et al., 1997). Compared with control plots, plants increased the relative abundances of bacterial genera such as *Sphingomonas, Arthrobacter*, and *Bryobacter*. Of these, the relative abundances of *Sphingomonas* and *Bryobacter* were highest in the alfalfa treatment. *Bryobacter* species can promote soil carbon cycling, and *Sphingomonas* is a rich microbial resource with strong degradative functions with great application potential in environmental protection (Du et al., 2017; Wang et al., 2022). *Arthrobacter* was most abundant in the tall wheatgrass treatment; this genus is strongly stress resistant, can efficiently degrade environmental pollutants, and is involved in biological N fixation (Li et al., 2017).

### 4.3 Effect of cultivating three salt-tolerant plants on interspecies relationships and microbial community stability

With increased soil nutrient contents and decreased EC in different plant treatments, the relative abundances of plant pathogenic fungi such as *Neocosmospora* and *Gibellulopsis* increased significantly, especially in the alfalfa treatment. Halophytes are vulnerable to infections caused by fungi and fungi-like pathogens, which may arise in their natural habitats or during various stages of the production process (Calabon et al., 2021). Some species of the genus *Gibellulopsis* cause wilt diseases in sugar beet, spinach, and potato (Iglesias-Garcia et al., 2013; Yang et al., 2017). Many species of *Neocosmospora* are opportunistic plant pathogens that cause canker, stem, and root rot diseases, as well as sugarcane wilt (Guarnaccia et al., 2022; Perera et al., 2023). In some cases, salinity can negatively affect plant pathogens. High salt concentrations reduce mycelial production and conidial formation because of a negative osmotic potential, as well as toxic and nutritional effects (Dikilitas and Karakas, 2014). A decrease in salinity may increase the relative abundance of pathogenic fungi such as *Neocosmospora* and *Gibellulopsis*. Additionally, mineral fertilizers without additional carbon sources can alter fungal communities (Paungfoo-Lonhienne et al., 2015), weaken plant–microbe networks (Banerjee et al., 2019), and increase the potential risk of harm from some plant pathogens (Berg and Koskella, 2018). Based on our findings, certain crops should not be planted in saline–alkaline soils that have been modified for agricultural purposes using certain plant species. For example, planting potatoes and sugarcane in soils previously cultivated with alfalfa is not recommended because of the increased relative abundance of *Gibellulopsis* and *Neocosmospora*.

Although soil factors can affect the abundance of pathogens, nutrient-induced enrichment of specific microbes can also impact plant health by directly antagonizing or indirectly regulating pathogens through the soil microbial community (Zeng and Zhuang, 2023). Plant-associated bacteria can be natural antibacterial agent (Fan et al., 2023). Plant selection or root filtering (Fan et al., 2018; Schmidt et al., 2019) occurs through the secretion of specific bioactive molecules that directly inhibit particular microbial taxa (Carrión et al., 2019), thereby achieving biocontrol of phytopathogens (Elsayed et al., 2022). The increased relative abundance of *Alternaria* and *Podospora* in alfalfa and tall wheatgrass treatments can exert antagonistic effects against fungal pathogens (Xu et al., 2012). In the chicory treatment, negative correlations between the fungal pathogens *Gibellulopsis* and *Neocosmospora* and other fungal microbes increased, suggesting that the presence of other fungal genera in the soil may inhibit pathogen growth and spread in soil. Additionally, specific bacteria in chicory cultivation exhibit high antagonistic activity against plant pathogens (Patkowska and Konopiński, 2016), thus helping to protect plants from pathogen attacks. We also found that the relative abundance of *Paraphoma*—a genus that can produce secondary metabolites with inhibitory and antibacterial capabilities, which can combat pathogenic fungi (Li et al., 2018)—was highest in the chicory treatment. Compared with saline–alkaline bare land, the interactions between bacteria and fungi in each treatment increased significantly. Decreased environmental stress (salt stress and nutrients) contributed to more complex microbial co-occurrence networks (Naher et al., 2018; Yan et al., 2017). The complexity of microbial co-occurring networks arises from the increased interactions between fungi and bacteria. Fungal and bacterial communities that coexist tend to be more stable than those dominated by either fungi or bacteria alone (Ushio et al., 2013), minimizing disturbances caused by environmental changes or pathogen invasion, especially in stressful environments (Wang et al., 2020).

## 5 Conclusions

We compared the effects of cultivating three salt-tolerant plant species over a 4-year period on severely saline–alkaline soils, and describe the potential roles of their rhizosphere microorganisms. While cultivation of none of these plant species significantly improved soil pH (compared with bare saline–alkaline land), each significantly reduced soil EC values and increased soil bacterial and fungal richness and diversity. Of the three plant species, chicory resulted in the most significant increases in SOM, AN, AK, and AP; *SQI* values, with levels of increase being greatest in chicory, then alfalfa, followed by tall wheatgrass, and finally, the control. Microbial community structures differ markedly between the control and treatment groups, showing a strong and significant correlation with *SQI*. The increased relative abundance of beneficial bacteria in treatments enhanced soil fertility, and beneficial fungi improved pathogen resistance. Over 4 years, the abundance of fungal pathogens like *Neocosmospora* and *Gibellulopsis* increased, especially in the alfalfa treatment. The chicory treatment had more negative correlations between fungal pathogens and microorganisms, inhibiting pathogen growth and spread. Future research could explore the effects of different organic fertilizers and salt-tolerant plant species on soil microorganisms to ameliorate heavily saline–alkaline soils. Additionally, evaluating the impact and economic feasibility of implementing these methods on a larger scale would provide valuable insights for more sustainable agricultural practices.

## Data Availability

The metagenomic sequences for this study have been deposited with NCBI under accession numbers: PRJNA1139873 (bacteria) and PRJNA1139872 (fungi).
