# Supplementary material for: Effects of microbial communities during the cultivation of three salt-tolerant plants in saline-alkali land improvement
Source: Front Microbiol. 2024 Oct 31;15:1470081. doi: 10.3389/fmicb.2024.1470081 (PMC11560748; doi:10.3389/fmicb.2024.1470081)
Supplement: Supplementary file 1 [file Data_Sheet_1.docx]

**Supplemental Figure**


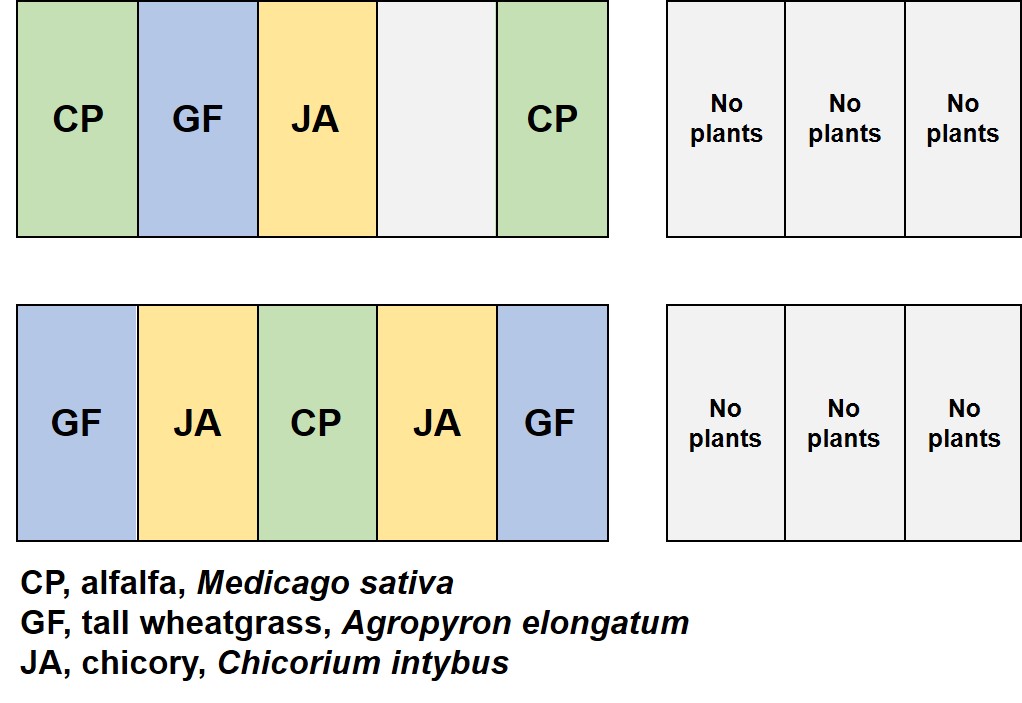


**Figure 1. Map of the experimental plot**

**Supplemental Tables**

**Table S1** Principal component contribution rates and weights of soil quality indicators

| Soil Quality Indicator | First principal component | | Second principal component | | Third principal component | | Fourth principal component | |
| --- | --- | --- | --- | --- | --- | --- | --- | --- |
|  | Loading | Weight | Loading | Weight | Loading | Weight | Loading | Weight |
| AN | 0.234 | 0.037 | 0.936 | 0.271 | 0.185 | 0.063 | −0.011 | 0.007 |
| SOM | 0.250 | 0.040 | 0.884 | 0.256 | 0.337 | 0.114 | 0.078 | 0.049 |
| AP | 0.967 | 0.153 | 0.072 | 0.021 | 0.207 | 0.070 | −0.058 | 0.036 |
| AK | 0.229 | 0.036 | 0.255 | 0.074 | 0.886 | 0.300 | −0.197 | 0.124 |
| pH | 0.123 | 0.019 | −0.204 | 0.059 | −0.928 | 0.314 | −0.183 | 0.115 |
| EC | −0.936 | 0.148 | −0.217 | 0.063 | 0.090 | 0.030 | 0.198 | 0.124 |
| Bacteria Chao | 0.938 | 0.148 | 0.168 | 0.049 | 0.096 | 0.033 | 0.022 | 0.014 |
| Bacteria Shannon | 0.831 | 0.131 | 0.240 | 0.070 | −0.039 | 0.013 | 0.431 | 0.270 |
| Fungi Chao | 0.922 | 0.146 | 0.324 | 0.094 | −0.052 | 0.018 | −0.078 | 0.049 |
| Fungi Shannon | 0.906 | 0.143 | 0.152 | 0.044 | 0.138 | 0.047 | 0.339 | 0.213 |
| Explained variance | 52.374 |  | 20.300 |  | 18.774 |  | 4.283 |  |
| Cumulative explained variance | 52.374 |  | 72.674 |  | 91.448 |  | 95.731 |  |

**Table S2** Membership values of each soil index under different treatments

| Treatments | CK | GF | JA | CP |
| --- | --- | --- | --- | --- |
| AN | 0.17 | 0.01 | 0.87 | 0.57 |
| SOM | 0.15 | 0.08 | 0.74 | 0.39 |
| AP | 0.03 | 0.96 | 0.94 | 0.70 |
| AK | 0.30 | 0.43 | 0.95 | 0.02 |
| pH | 0.37 | 0.28 | 0.61 | 0.05 |
| EC | 0.31 | 0.98 | 0.97 | 0.99 |
| Bacteria chao | 0.07 | 0.75 | 0.81 | 0.68 |
| Bacteria Shannon | 0.41 | 0.94 | 0.98 | 0.96 |
| Fungi chao | 0.23 | 0.84 | 0.95 | 0.94 |
| Fungi Shannon | 0.31 | 0.90 | 0.94 | 0.83 |

**Table S3** Number of linear links related to different species in networks for different treatments

|  | CK | | GF | | JA | | CP | |
| --- | --- | --- | --- | --- | --- | --- | --- | --- |
|  | Pos | Neg | Pos | Neg | Pos | Neg | Pos | Neg |
| Alternaria | 4 | 7 | 7 | 4 | 32 | 4 | 7 | 6 |
| Gibellulopsis | 0 | 0 | 4 | 3 | 5 | 7 | 11 | 5 |
| Neocosmospora | 7 | 0 | 6 | 9 | 8 | 14 | 27 | 3 |
| Paraphoma | 0 | 0 | 2 | 2 | 5 | 3 | 4 | 1 |
| Podospora | 0 | 0 | 7 | 3 | 6 | 5 | 21 | 6 |
| Talaromyces | 22 | 52 | 13 | 14 | 13 | 2 | 15 | 9 |
